# Transcriptomic Profiling Analysis of *Arabidopsis thaliana* Treated with Exogenous *Myo*-Inositol

**DOI:** 10.1371/journal.pone.0161949

**Published:** 2016-09-07

**Authors:** Wenxing Ye, Weibo Ren, Lingqi Kong, Wanjun Zhang, Tao Wang

**Affiliations:** 1 Department of Grassland Science, China Agricultural University, Haidian District, Beijing, PR China; 2 State Key Laboratory of Agro-biotechnology, China Agricultural University, Haidian District, Beijing, PR China; 3 Beijing Key Laboratory of Grassland Science, China Agricultural University, Haidian District, Beijing, PR China; 4 Institute of Grassland Research of Chinese Academy of Agricultural Science, Saihan District, Hohhot, Inner Mongolia, PR China; Institute of Genetics and Developmental Biology Chinese Academy of Sciences, CHINA

## Abstract

*Myo*-insositol (MI) is a crucial substance in the growth and developmental processes in plants. It is commonly added to the culture medium to promote adventitious shoot development. In our previous work, MI was found in influencing *Agrobacterium*-mediated transformation. In this report, a high-throughput RNA sequencing technique (RNA-Seq) was used to investigate differently expressed genes in one-month-old *Arabidopsis* seedling grown on MI free or MI supplemented culture medium. The results showed that 21,288 and 21,299 genes were detected with and without MI treatment, respectively. The detected genes included 184 new genes that were not annotated in the *Arabidopsis thaliana* reference genome. Additionally, 183 differentially expressed genes were identified (DEGs, FDR ≤0.05, log_2_ FC≥1), including 93 up-regulated genes and 90 down-regulated genes. The DEGs were involved in multiple pathways, such as cell wall biosynthesis, biotic and abiotic stress response, chromosome modification, and substrate transportation. Some significantly differently expressed genes provided us with valuable information for exploring the functions of exogenous MI. RNA-Seq results showed that exogenous MI could alter gene expression and signaling transduction in plant cells. These results provided a systematic understanding of the functions of exogenous MI in detail and provided a foundation for future studies.

## Introduction

*Myo*-inositol (MI) is a small molecule that is important in many different developmental and physiological processes in eukaryotic cells [1, 2]. MI participates in the phosphatidylinositol (PtdIns) signaling pathway, auxin storage and transport, phytic acid biosynthesis, cell wall biosynthesis, and the production of stress-related molecules [3, 4]. MI is commonly added in plant culture media, as its addition is believed to improve plant regeneration [5–7]. Because of its important function in many eukaryotic pathways, inositol and its derivatives have been extensively studied.

In plants, there are seven naturally occurring isomers of inositol, MI is the most abundant form in biological systems [8]. In plant cells, MI is synthesized from glucose by enzymes L-*myo*-inositol 1-phosphate synthase (MIPS) and inositol monophosphate phosphatase (IMPase) [4, 9]. In *Arabidopsis*, *MIPS1* is a member of MIPS gene family (*MIPS1*, *MIPS2* and *MIPS3*) and contributes to the major pool of MI, which is associated with physiological responses to salt and abscisic acid [8]. The *atmips1* mutant was significantly different from the wild type plant in growth and development. However, the defect of *atmips1* can be rescued by exogenous MI [10].

The MI oxidation pathway (MIOP) effectively consumes MI and is an important route for cell wall polysaccharide synthesis. In this pathway, *myo*-inositol oxygenase (MIOX) is a key enzyme in the UDP-D-glucuronic acid (UDP-GlcA) synthesis process [11]. UDP-GlcA is an important precursor for cell wall synthesis and the production of hemicellulose precursors [4, 12]. In addition, another oxidation pathway can also generate UDP-GlcA—the sugar nucleotide oxidation pathway (SNOP) [13]. *MIOX2* is a member of the MIOX gene family in *Arabidopsis* (*MIOX1*, *MIOX2*, *MIOX4*, *MIOX5*) [14], and *miox2* mutants exhibit a defect in cell wall biosynthesis [15]. It has been reported that MIOX might also be involved in the production of ascorbate [16] and, consequently, protection from ROS-mediated injury [17]. Ma et al. [18] demonstrated that the expression of the *MIOX2* and *MIOX4* genes can be stimulated by the transcription factor bZIP11 under sugar starvation [18]. This indicates that MIOX might be activated under sugar starvation conditions to generate alternative sugar sources, thereby indirectly contributing to metabolic homeostasis. Some research results indicated MI translocation from the shoot to the root via phloem provides information on the shoot photosynthetic status to the root [14, 19]. In recent years, it has been reported that MI metabolism is implicated in plant programmed cell death (PCD). MI was involved in the generation of reactive oxygen species (ROS), such as H_2_O_2_, and regulated defense gene expression [20–23]. Some MI derivatives also involved PCD and stress, such as ceramide and phosphoinositide-specific phospholipase C (PLC) [24, 25]. *Arabidopsis* mutants *atmips1* and *atmips2* were more susceptible to bacterial, viral, and fungal pathogens. In addition, MI and MI derivatives were involved in intracellular Ca^2+^ release [26], chromatin remodeling, mRNA export, regulation of telomere length and DNA repair [26–28]. Not only MI but also its derivatives are important in various processes in plant cells.

Previous reports suggested that exogenous MI affected plant responses to salt stress, *Agrobacterium*-mediated transformation and gene expression. Salt-induced injury to the chromosomal apparatus can be ameliorated by exogenous inositol [29]. However, without supplementation of MI in the culture medium, the calluses were more amenable to *Agrobacterium*-mediated transformation of perennial ryegrass and rice [30]. The results suggested that exogenous inositol could affect the physiological status of plant cells. We are interested in identifying gene expression that was significantly affected by exogenous MI supplementation. Thus affected the sensitivity of plant cells to *Agrobacterium* infection and improving the transgenic efficiency in the long run.

RNA sequencing (RNA-Seq) represents the latest and most powerful tool for characterizing the transcriptome [31]. In the past few years, there have been many reports using RNA-Seq data to represent integrated networks of the cellular biology in various plants [32, 33].

In this study, the analysis of gene expression was performed in *Arabidopsis thaliana* after exogenous MI treatment. Comprehensive information regarding the effects of exogenous MI on gene expression and physiological metabolism was presented, which provided insight into the molecular effects of MI on plant cell sensitivity to *Agrobacterium* infection. This work also provided an integrated understanding of the function of exogenous MI in growth and development of plants.

## Materials and Methods

### Plant materials and growth conditions

Following the procedure of *Agrobacterium* transformation *Arabidopsis thaliana* roots [34], seeds of wild-type *Arabidopsis thaliana* (Col-0) were planted in B5 medium [6] supplied with or without MI (MI+ or MI-). The concentration of MI added in the medium was 100 mg l^-1^. Seeds were stratified at 4°C for 2 d in darkness and then were transferred to a phytotron set at 23°C under a 16-h light/8-h dark photoperiod (light intensity 120 μmol m^-2^ s^-1^) for germination and further growth. After four weeks, the seedlings were collected for RNA extraction.

### RNA isolation and Illumina sequencing

Total RNA was extracted using the TRIzol^®^ reagent (Invitrogen) according to the manufacturer’s protocol. Two biological replicates were used for each sample, and each sample contained tissues from at least ten whole plants (shoots and roots) grown on medium supplied with MI+ or MI- treatment. Poly (A)^+^ RNA was purified from 5 μg of pooled total RNA using oligo (dT) magnetic beads, sheared into short fragments, and primed for cDNA library synthesis using the TruSeq RNA sample preparation kit according to the manufacturer’s instructions (Illumina). After quantitation using a Library Quantification Kit (Illumina GA Universal, Kapa, KK4824), the samples were clustered (TruSeq paired-end cluster kit v3-cBot-HS; Illumina). Finally, the four libraries were sequenced using an Illumina HiSeq2500^™^ platform with a read length of 100 bp (PE100, paired-end).

### RNA-Seq data pre-processing and quality control

To obtain high quality reads, raw pair-end reads were evaluated, and low quality reads and rRNA sequences were removed by software. First, the first 10 bases from raw reads were removed due to unstable performance in the sequence component analysis using FASTX-toolkit pipeline version 0.0.13 (http://hannonlab.cshl.edu/fastx toolkit/). Second, low quality reads were removed to ensure that more than 80% of the bases of each reserved read possessed Illumina Quality >30 (Q30 indicating 0.1% sequencing error rate). Last, sequencing quality of the reads was examined by FastQC software (V0.10.1) (http://www.bioinformatics.babraham.ac.uk/projects/fastqc/). The filtered reads are clean reads. The raw sequence data in this test have been uploaded to NCBI (http://www.ncbi.nlm.nih.gov/bioproject/), and the accession number is PRJNA296761.

### Mapping reads to the reference genome and annotated genes

Clean reads were mapped to the *A*. *thaliana* genome (downloaded from www.phytozome.net) by TopHat Version 2.0.10 (http://tophat.cbcb.umd.edu/) utilizing Bowtie 2 version 2.1.0 (http://bowtiebio.sourceforge.net/bowtie2/index.shtml) with the following parameters: both maximum splicing mismatches and maximum read mismatches were no more than 2; maximum intron length allowed to be 2,000. Mapping results generated by TopHat were filtered to retain the only unique mapped reads before being piped into Cuffdiff to estimate read counts for each gene. FPKM (fragments per kilobase of exon per million fragments mapped) values were calculated by an in-house script based on the count table of Cuffdiff output, using at least 2 replicates with correlation coefficients of >0.93 in each library. The length of the longest transcript was taken as the length of the gene during the calculation. Genes were annotated with annotation file Ath_AGI_LOCUS_TAIR10_Aug2012 [downloaded from MapMan website [35]. Novel genes were excavated by Cufflinks (http://cufflinks.cbcb.umd.edu/) assembly reads compared with known gene models in the *A*. *thaliana* genome.

### Identification of GO categories

The Blast2GO program [36] was used to obtain GO annotation of the unigenes. The WEGO software was then used to perform GO functional classification of all unigenes to view the distribution of gene functions. To statistically analyze GO-term enrichment, Blast2GO integrated the Gossip package for statistical assessment of differences in GO-term abundance between two sets of sequences [37]. This package uses Fisher’s exact test and corrects for multiple testing. A one-tailed Fisher’s exact test was carried out using a false discovery rate (FDR) with a filter value of <0.05. Blast2GO returns GO terms over-represented at a specified significance value [38].

### Analysis and detection of differentially expressed genes (DEGs)

In this study, FPKM was used to measure the gene expression in RNA-Seq. Genes with differential expression between these two groups were identified using the R package edgeR. (http://www.bioconductor.org/packages/release/bioc/html/edgeR.html). The results of all statistical tests were corrected for multiple testing with the Benjamini–Hochberg false discovery rate (FDR) of adjusted *p* value ≤0.05, and there was at least a two-fold change (≥1 or ≤− 1 in log_2_ ratio value) in FPKM between two libraries. For functional annotation purposes, genes showing significant expression changes in response to MI treatment were analyzed using the DAVID system [39] and TAIR (http://www.arabidopsis.org/tools/bulk/go/index.jsp) databases. The KEGG Orthology Database (http://www.genome.jp/kegg/ko.html) was used for pathway mapping [40]. We used the MapMan package (http://MapMan.gabipd.org) to visualize the metabolic pathways of the DEGs [35].

### Quantitative real-time RT-PCR analysis

To confirm the RNA-Seq results, fifteen genes were randomly selected to test gene expression levels using real-time qPCR. Primer sets were designed with the Primer Premier 5.0 software (S8 Table). Reactions contained 10 μl total volume, 0.2 μl of reverse and forward primers, 3.6 μl of ddH_2_O, and 5 μl of the PCR master mix [SYBR^®^ *Premix Ex Taq* (Tli RNaseH Plus), TaKaRa]. The following PCR program was used: 95°C for 30 s, followed by 40 cycles of 95°C for 5 s and then 60°C for 30 s. The melting curve generation (55–95°C) following the final cycle of the PCR was performed to test the specificity of the PCR amplification. Using the Eco^™^ Real-Time PCR System (Illumina), the relative expression level of each gene was calculated by the 2^-ΔΔCT^ method using the *AtUBQ5* or *AtElf4α* as an internal reference gene [41]. All samples were run in triplicate in separate tubes, and each cDNA sample was run in duplicate. All data were presented as the mean ± SD after normalization. To compare changes in of genes relative expression (GRE) in qRT–PCR tests and RNA-Seq data, Log_2_GRE and log_2_FC of RNA-Seq (FC came from FPKM of a gene expression) was presented.

## Results

In the experiments, *Arabidopsis thaliana* Col-0 seeds were germinated on medium supplied without or with MI at five different concentrations (S1A and S1B Fig). We found the primary root length of 4-week-old seedlings were significantly different among treatments. The roots of *Arabidopsis* seedling grown on the medium supplied without and with MI at 0, 50, 100, 500, and 1000 mg l^-1^ were 4.3, 3.3, 3.1, 2.5 and 3.0 cm, respectively (S1A and S1C Fig). Leave color was slightly different under 500 and 1000 mg l^-1^ MI concentration conditions compared with other treatments, and there were some yellow leaves on plants at 500 and 1000 mg l^-1^ MI concentrations. But no noteworthy of changes on shoots were found under other treatments with different MI concentration. The roots of 4-week-old seedlings were collected and infected with wild type *A*. *tumefaciens* strain A208, and crown galls were counted to determine the sensitivity of explants to *Agrobacterium* transformation. The results showed that tumorigenesis efficiency in the roots grown with the addition of exogenous MI was significantly higher than the control with no MI addition except 1000 mg l^-1^ MI concentration (S1B and S1D Fig). In addition, compared with no MI, the formation of increased lateral roots and root hairs was observed in the treatment of 100 mg l^-1^ MI concentration (S2 Fig). The results suggested that the exogenous MI affected roots growth and enhanced the sensitivity of *Arabidopsis* roots to *Agrobacterium* transformation. The transformation efficiency was the highest in 100 mg l^-1^ MI treatment. We hypothesized that these differences might be caused by exogenous MI regulated the expression of genes in plants. It is of interest to note how the genes were differently expressed and also affected the metabolic pathways. Here, we analyzed the gene transcription level of the whole plant with (MI+ represented 100 mg l^-1^ MI condition) or without (MI- represented 0 mg l^-1^ MI condition) MI treatments by the method of RNA-Seq, to confirm our hypothesis.

### Transcriptome sequencing and alignment to the reference genome

To comprehensively analyze the molecular mechanisms and the genome-wide transcriptional responses induced by exogenous MI supplementation, we sequenced two cDNA libraries taken from *Arabidopsis thaliana* wild-type (Col-0) treated with or without MI (MI+ or MI-), named A1 (MI-) and A2 (MI+). As a result, we obtained approximately 51 million sequences reads by Illumina HiSeq^™^ 2500 sequencing with PE100. To assess the accuracy of the data analysis, we filtered the original sequences to ensure the quality of each read and removed read values less than twenty bases (no more the 20%), sequences with N content less than 5%, and rRNA sequences. Finally, there were 40.3 million high quality reads, and more than 95.7% clean reads were mapped to the *Arabidopsis* genome (https://www.arabidopsis.org/) using TopHat2. In the clean reads, 60.31%-60.55% were perfectly matched and 6.33–6.06% had no more than two base mismatches, while unmapped reads were 3.72–4.22% (Table 1).

**Table 1:** Reads number based on the RNA-Seq data in two libraries of *A*. *thaliana* wild-type (Col-0) under exogenous *myo*-inositol (MI+ or MI-).

	A1(MI-)	A2(MI+)
**Total clean reads**	19256956	21043070
**Mapped reads**	18541170(96.28%)	20154886(95.78%)
**Perfect match reads**	11183518(60.31%)	12203171(60.55%)
**≤2 bp mismatch reads**	1174445(6.33%)	1222375(6.06%)
**Unmapped reads**	715787(3.72%)	888184(4.22%)

Clean reads which are filtered from dirty raw reads are mapped to reference sequences using TopHat. Mismatches no more than 2 bases are allowed in the alignment. Mapped reads are the sum of perfect match reads and less than 2 bp mismatch reads. Numbers enclosed in parenthesis represent the percents of reads in each library.

By comparison with the *Arabidopsis* genome, all 51 million reads were assembled into 26,442 genes with TopHat2 and Cufflinks, and the estimates were made of expression levels (with FPKM quantitative gene expression level). Among the two treatments, a total of 21,868 genes with a FPKM (fragments per kilobase of exon per million fragments mapped) value >0 was found for A1 and A2, and the number of genes for each treatment was 21,299 (A1) and 21,288 (A2) (S1 Table). It is worth noting that 184 new genes were detected by Cufflinks while some genes were filtered out because the coding sequence was too short (less than 50 amino acids) or it only contained a single exon (S2 Table). In addition, size distribution of the 21,868 genes is shown in Table 2. The genes were divided into five groups based on sizes. The most abundant group was the 1001–1,500 bp group (5334; 24.4% of all 21,868 genes) and the no less than 2,000 bp group (5,588; 25.6% of all 21,868 genes), followed by 501–1,000 bp (4967; 22.7% of all 21,868 genes) and 1,501–2,000 bp (3,859; 17.6% of all 21,868 genes), and 78–500 bp (2120; 9.7% of all 21,868 genes) was a relatively small group.

**Table 2:** Distribution of all 21868 genes detected in *Arabidopsis* with exogenous MI treatments via RNA-Seq technology.

Gene length (bp)	number	Percentage (%)
**78–500**	2120	9.7
**501–1000**	4967	22.7
**1001–1500**	5334	24.4
**1501–2000**	3859	17.6
**≥2001**	5588	25.6
**Total**	21868	100

All clean reads were assembled into 21868 genes. The size distribution can be divided into five species. The total number and percentage of all genes are presented in the table.

### RNA-Seq global data analysis and evaluation of differential gene expression

**Fig 1:**
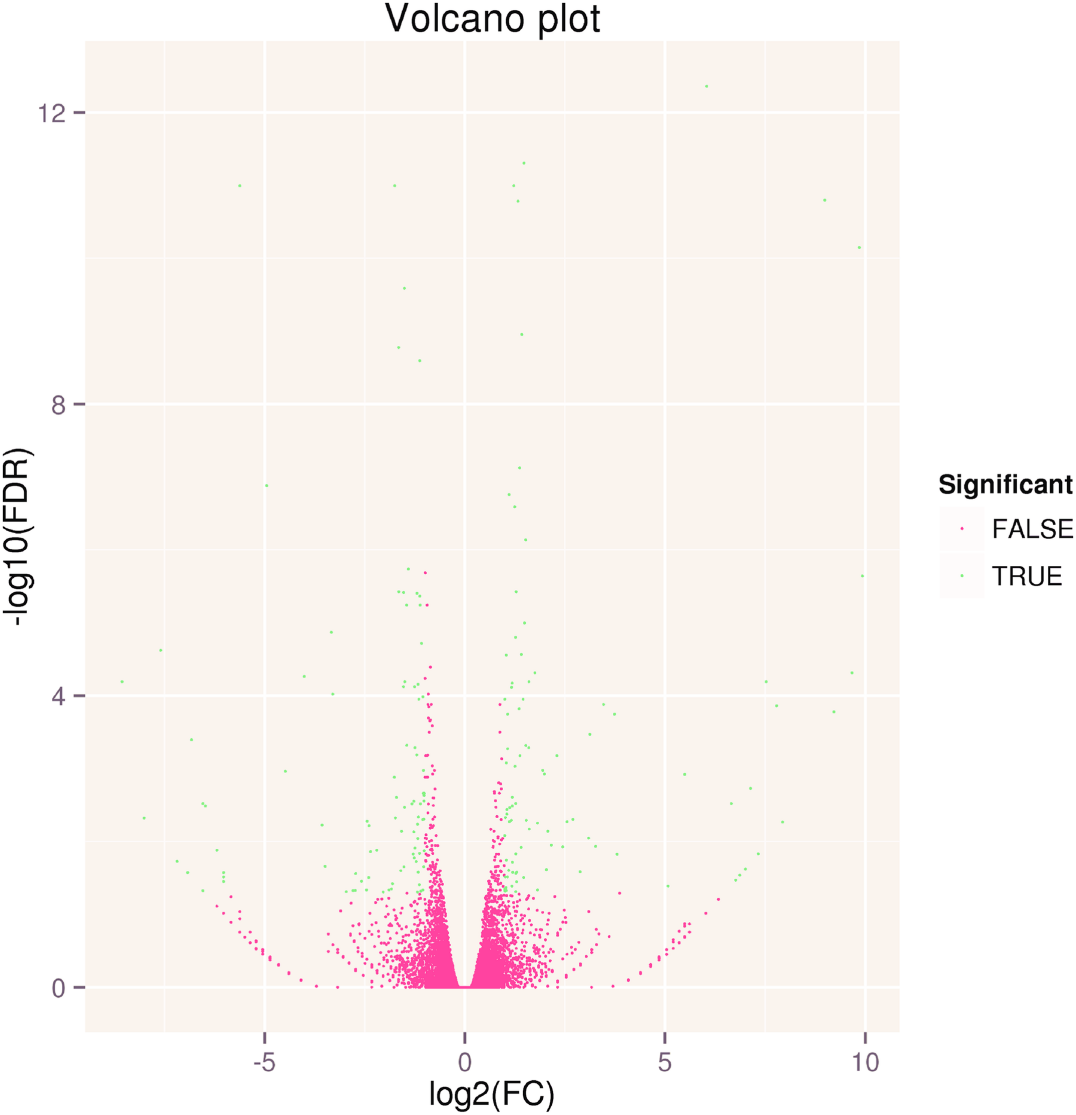
Volcano plot of RNA-Seq data. A volcano plot is a scatter plot that is often used when analyzing micro-array data sets to provide an overview of interesting genes. The log_2_ (FC) (fold change) is plotted on the x-axis, and the negative log_10_ (FDR) (*p*-value) is plotted on the y-axis. The red point shows no differential gene expression with the absolute value of log_2_ (FC) less than 1 (FC = 2) and FDR no less than 0.05. The blue point show differentially expressed genes with the absolute value of log_2_ (FC) no less than 1 (FC = 2) and FDR less than 0.05.

To provide an overview of interesting genes, a volcano plot was used to show the overall distribution of all DEGs. In the volcano plot, the green dots represent significant differentially expressed genes, and the red dots represent gene expression that was not significantly different (Fig 1).

**Fig 2:**
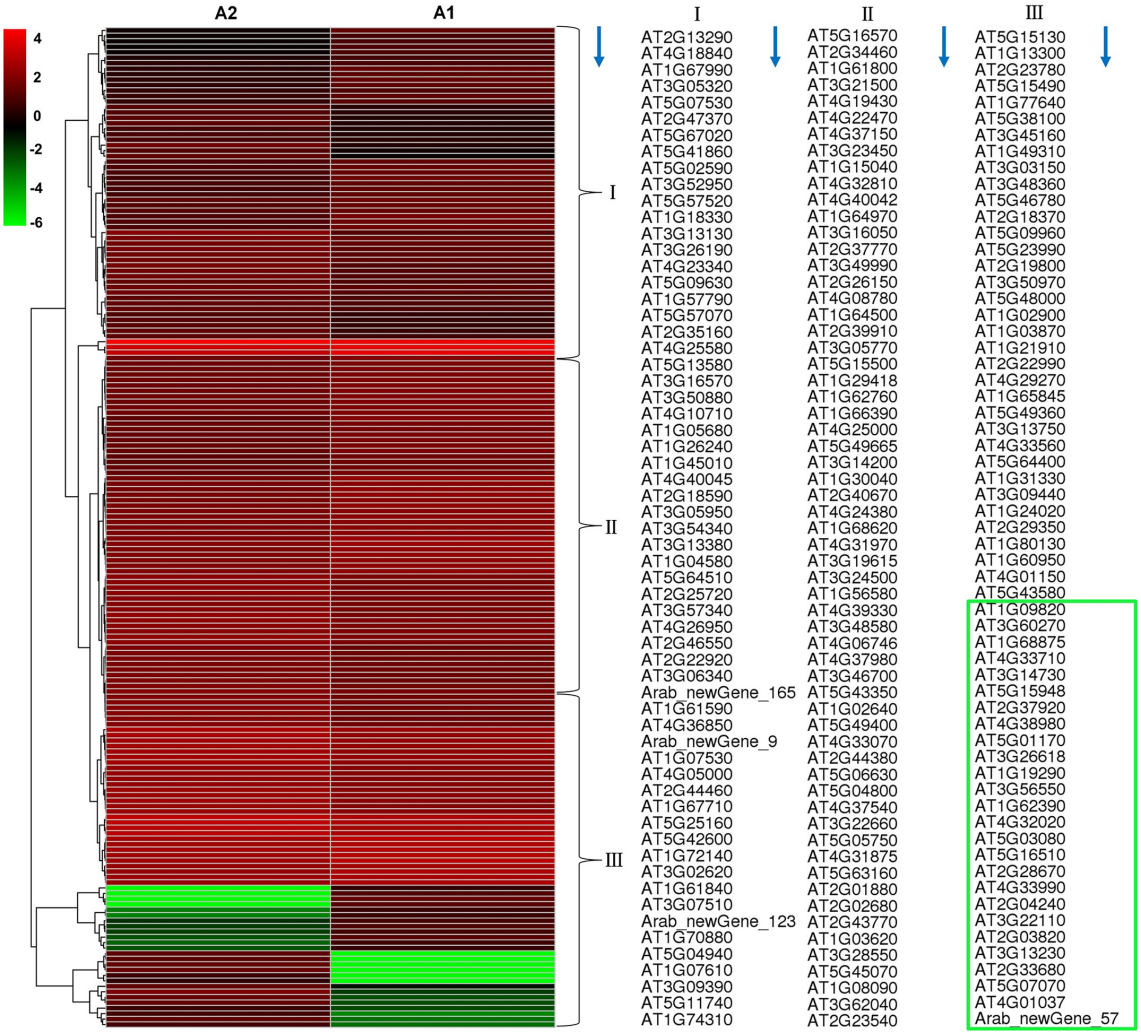
Hierarchical cluster analyses of gene expression based on log ratio RPKM data. The cluster display expression patterns for a subset of DEGs in two comparisons (A1-vs-A2) between two treatments. The *color key* represents RPKM normalized log_10_ transformed counts. *Red* represents high expression, *green* represents a low expression. *Each column* represents an experimental condition, and *each row* represents a gene. The columns are evenly divided into three groups, I, II and III. Each group contains 61 genes, their order are arranged in accordance with the blue arrow direction. The green box contains 26 genes, which represented the green rows in III group.

To identify the DEGs between the two groups, we used the edgeR method to find the DEGs between these two samples (A1 and A2). The DEGs were defined as the fold change of FPKM expression values and were at least 2 in either direction when the log_2_ ratio (log_2_ fold change) ≥1 and the false discovery rate (FDR) ≤0.05. A total of 183 DEGs were detected between A1and A2 (Fig 2; S3 Table). Of them, 93 genes were up-regulated and 90 genes were down-regulated, indicating that exogenous MI affected transcription of *Arabidopsis thaliana*. To gain further insight, the DEGs were separated into three groups based on fold change: 2-4-fold (1 ≤log_2_ ratio <2, FDR ≤ 0.05), 4-10-fold (2 ≤log_2_ ratio<3.32, FDR ≤ 0.05) and more than 10-fold (log_2_ ratio≥3.32, FDR≤ 0.05) (Table 3). Among the DEGs, there were 119 genes at 2-4-fold (included 62 genes that were up-regulated and 57 genes that were down-regulated), 24 genes at 4-10-fold (included 11 up-regulated genes and 13 down-regulated genes), and the rest of the 40 genes were greater than 10-fold (both up-regulated and down-regulated were twenty) (Table 3). The up-regulated and down-regulated gene numbers were almost the same in DEGs from the two samples. Four new genes were all up-regulated.

**Table 3:** All 183 DEGs were divided according to the folds of FPKM value between two treatments.

Fold	A1_vs_A2		
	Number	Up-regulated	Down-regulated
**2≤fold<4**	119	62	57
**4≤fold<10**	24	11	13
**Fold≥10**	40	20	20
**Total number**	183	93	90

Base on the folds of FPKM value between two treatments, all 183 DEGs can be divided into three groups.

### Hierarchical clustering of DEGs between two treatments

Clustering analysis was used to determine differences in gene expression patterns of the groups treated with or without MI. By clustering, the similar gene expression patterns were classified together to show the relevance of gene functions. To identify clusters with functional enrichment, we performed hierarchical clustering of the expression patterns of 183 DEGs, and Pearson correlation was used to determine the distance metric for gene expression patterns with functional enrichment. The results were shown in Fig 2. Log ratio values (FPKM) were used for gene expression analyses. The hierarchical clustering analysis indicates that the numbers of the down-regulated transcripts and up-regulated transcripts are not different. However, we observed clear differences in the expression levels of 26 genes (Fig 2, shown in the green rows.), including 6 genes encoding pentatricopeptide repeat-containing protein (PPRs), which had a close relationship in the hierarchical cluster plot. The related DEGs require further analysis to study their biological pathways and functions.

### Gene ontology (GO) analysis of RNA-Seq data

**Fig 3:**
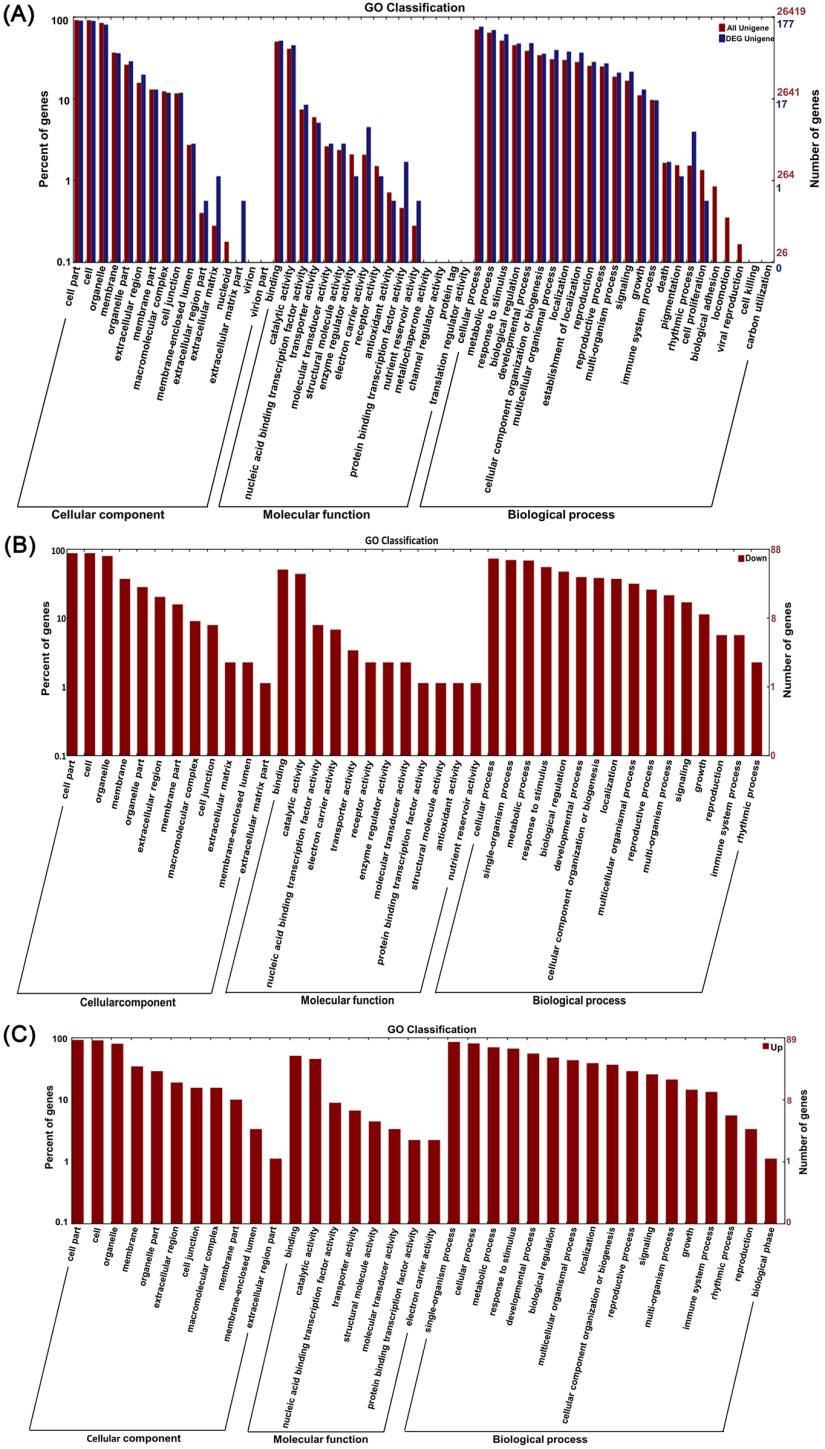
GO classifications of genes. The results are summarized in three main categories: biological processes, molecular functions and cellular components by GO analysis. (A) GO classifications of all genes between the two treatments and all 177 DEGs between the two treatments. **(B)** GO analysis of the down-regulated genes in A1-vs-A2. **(C)** GO analysis of the up-regulated genes in A1-vs-A2.

To obtain a visualized data set, GO enrichment analysis was performed. By GO analysis, all genes were divided into three major groups—biological processes, cellular components and molecular function. Using BLAST2GO (version 2.3.5) (http://www.blast2go.org/), a total of 5256 GO terms were associated with all 21,868 genes and were classified into 56 annotated functional subcategories (Fig 3A). Biological processes comprised the majority of the GO annotations (3322; 63.2%), followed by molecular functions (1439; 27.4%) and cellular components (495; 9.4%). In the three categories, nine subcategories were the most abundant sub-groups (each subcategory included no less than 10,000 genes): cell part (GO:0044464), cell (GO:0005623), organelle (GO:0043226), binding (GO:0005488), catalytic activity (GO:0003824), cellular process (GO:0009987), metabolic process (GO:0008152), response to stimulus (GO:0048583) and developmental process (GO:0003006). In theory, all genes were associated with at least one GO term. However, in fact, some genes were not annotated to any GO term. In the GO enrichment of DEGs, the 177 genes from all 183 DEGs were associated with 996 GO terms in *Arabidopsis* (Fig 3A). Biological processes included 678 GO terms (152 genes, 83.1% of all DEGs), molecular functions included 226 GO terms (124 genes; 67.8% of all DEGs) and cellular components included 92 GO terms (141 genes; 77.0% of all DEGs). The GO enrichment analyses of up-regulated and down-regulated genes of A1-v-A2 were shown in Fig 3B and 3C. Eighty-eight down-regulated genes (Fig 3B) and 89 up-regulated genes (Fig 3C) were respectively separated into 40 and 36 subcategories. Differences were found when we compared the up-regulated and down-regulated subcategories. For example, four subcategories were only found in molecular function categories of Fig 3B. They were receptor activity (GO:0004872, GO:0005484), antioxidant activity (GO:0004601), enzyme regulator activity (GO:0046910, GO:0004867) and nutrient reservoir activity (GO:0045735). The differences of function subcategories showed exogenous MI impacted the gene expression patterns. These data offered us some useful information to understand the gene expression and function in plant growth, development, metabolism and stress resistance. The results indicates that MI has a distinct effects on the growth and metabolisms of *Arabidopsis thaliana*.

### KEGG and MapMan pathway analyses

Analyzing the pathway annotations help to further interpret the biological functions of the differently expressed genes. The KEGG (Kyoto Encyclopedia of Genes and Genomes) is a major public pathway-related database. To perform pathway and functional classification of *Arabidopsis* DEGs induced by exogenous MI treatments, all DEGs were analyzed by mapping to the KEGG database. We obtained 32 pathways with differentially expressed genes assigned to KEGG orthologues (S4 Table, with 17 up-regulated genes and 15 down-regulated genes). By KEGG enrichment analysis, the 32 pathways were separated into four biological function classifications: genetic information processing, cellular processing, environmental information processing and metabolism processing (S3 Fig). Most of the pathways were related to metabolism processing. One example is the phenylpropanoid biosynthesis pathway, which is one of the most significant secondary metabolism pathways in plants [42]. Starch and sucrose metabolism, ribosome biogenesis, base excision repair and RNA transport are very important for carbohydrate metabolism and genetic information processing. Phenylpropanoid biosynthesis and starch and sucrose metabolism-related genes were reported to be associated with the formation of the cell wall [43, 44]. AT1G02640 (k05349) was noticed up-regulated with addition of MI (A2) and was assigned to the two pathways (Ko00940 and Ko00500). Its products are potentially targeted to the cell walls [45]. The *MIOX2* gene (AT2G19800; Ko00562) was also slightly up-regulated in the A2 sample, which was one of four *MIOX* genes in *Arabidopsis* (*MIOX1*: At1g14520, *MIOX2*: At2g19800, *MIOX4*: At4g26260, and *MIOX5*: At5g56640). MIOX converts MI to D-GlcUA and activates D-GlcUA into UDP-GlcUA in plant cells, which serves as an important sugar precursor for plant cell walls. Four *MIOX* genes were differentially regulated during development, and *MIOX2* gene was the predominant one [15]. In addition, we observed the expression level of *AtMIOX2* was upregulated 2.3 folds and 28 folds in roots and root calluses with MI treatment, respectively (S4A and S4F Fig). The result indicated that the expression levels of genes in roots were effected clearly by exogenous MI. *UGD3* (AT5G15490, Ko00500), encodes a UDP-glucose dehydrogenase (UGD), showed similar changes to *MIOX2*. The alpha-amylase 1 gene (AT4g25000, Ko00500) was down-regulated in the A2 sample. The results indicated that application of exogenous MI promoted the synthesis of cell wall precursors and reduced the hydrolysis of starch. The DEGs analysis didn’t identify any genes involved in inositol phosphate metabolism pathway. For example, *MIPS1*, *MIPS2*, *PIS1*, *PLC2*, *IPK2β*and *IPK1*, the key genes for biosynthesizing MI, inositol phosphates and phosphatidylinositol phosphates were not identified through the DEG analysis. Using qRT-PCR (S4E and S4I Fig), we found *AtMIPS1*and *AtMIPS2* were significantly lower in root calluses with MI+ treatment, but had no significant difference in roots. *PIS1*, *PLC2*, *IPK2β*and *IPK1* had no significant difference both in roots and root calluses under MI- and MI+ treatments. The results demonstrated that application of exogenous MI significantly affected the expression levels of MI biosynthesis genes.

**Fig 4:**
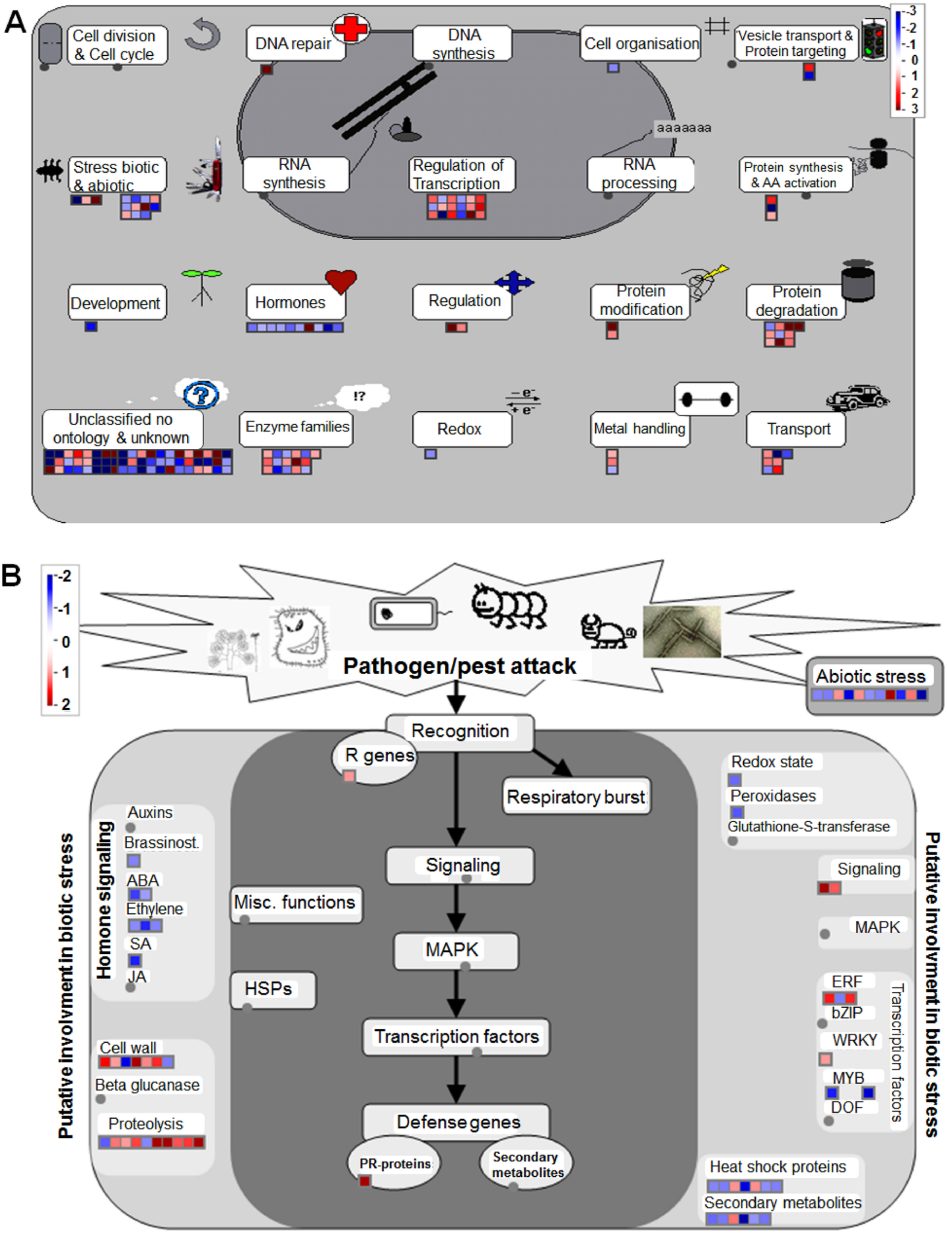
MapMan overview of cellular function (A) and biotic stress (B) showing all DEGs between the two treatments with exogenous *myo*-inositol. The *big grey circle* is an illustrated map of nucleus. The *small grey circle* indicate annotated biological process (metabolites). The *small squares* represent individual genes. The *color key* represents RPKM normalized log_2_ transformed counts. *Red* represents up-regulation and *blue* represents down-regulation between two treatments with exogenous *myo*-inositol.

In recent years, biological databases have grown rapidly. Different omics analysis systems have provided a better understanding of genes function. The MapMan system visualizes the DEGs more thoroughly in different pathways. In the experiment, 181 genes of all DEGs were subjected to MapMan tool analyses and were classified into 21 MapMan major pathways and 52 branch pathways, including 120 MapMan functional classes (S5 Table). In the 181 DEGs, there were 68 genes that were not assigned to functional classes. An overview of the 181 DEGs between A1 and A2 in terms of cellular function and biotic stress pathways was shown in Fig 4. The figure showed the gene related to particular biological processes of interested and displayed the log_2_ FPKM-normalized expression counts on the pictorial diagrams. In Fig 4A, there were ten genes associated with hormones metabolism, nine down-regulated and one up-regulated. Fourteen genes were associated with biotic stress (three genes) and abiotic stress (eleven genes). Regulation of transcription contained eighteen genes, and sixteen genes were associated with enzyme families. Through DEG analysis, we also identified two transcription factors, *AtSUVH1* (AT5G04940) and *AtSUVH5* (AT2G35160), both of them are involved in chromatin modification. Interestingly, the expressions of these two genes were upregulated both in roots and calluses (S4C, S4D, S4G and S4H Fig). The results were similar to the DEGs. Because of MI was associated with plant defense responses, we examined the DEGs involved in pathogen resistance. In Fig 4B, only two genes were assigned to biotic stress pathways. In addition, we used the DAVID system to analyze the GO terms of the DEGs for a better understanding of the functional classification and metabolic pathways, and the threshold of the *P* value was set to ≤0.05 for GO terms and the enrichment score value ≥ 0.80. Nine classes, such as oxidation-reduction, secondary metabolic process, and plant-type cell wall, were identified (S6 Table). For the biological process of oxidation reduction, *SAG13* (AT2G29350), is associated with senescence, was downregulated in A2. We had observed obvious browning of calluses on MI- treatment (S5A Fig), but not in MI+ treatment (S5B Fig), which indicated that exogenous MI could reduce cell death and maintain normal cell growth and development.

### Real-time quantitative PCR validate the RNA-Seq results

**Fig 5:**
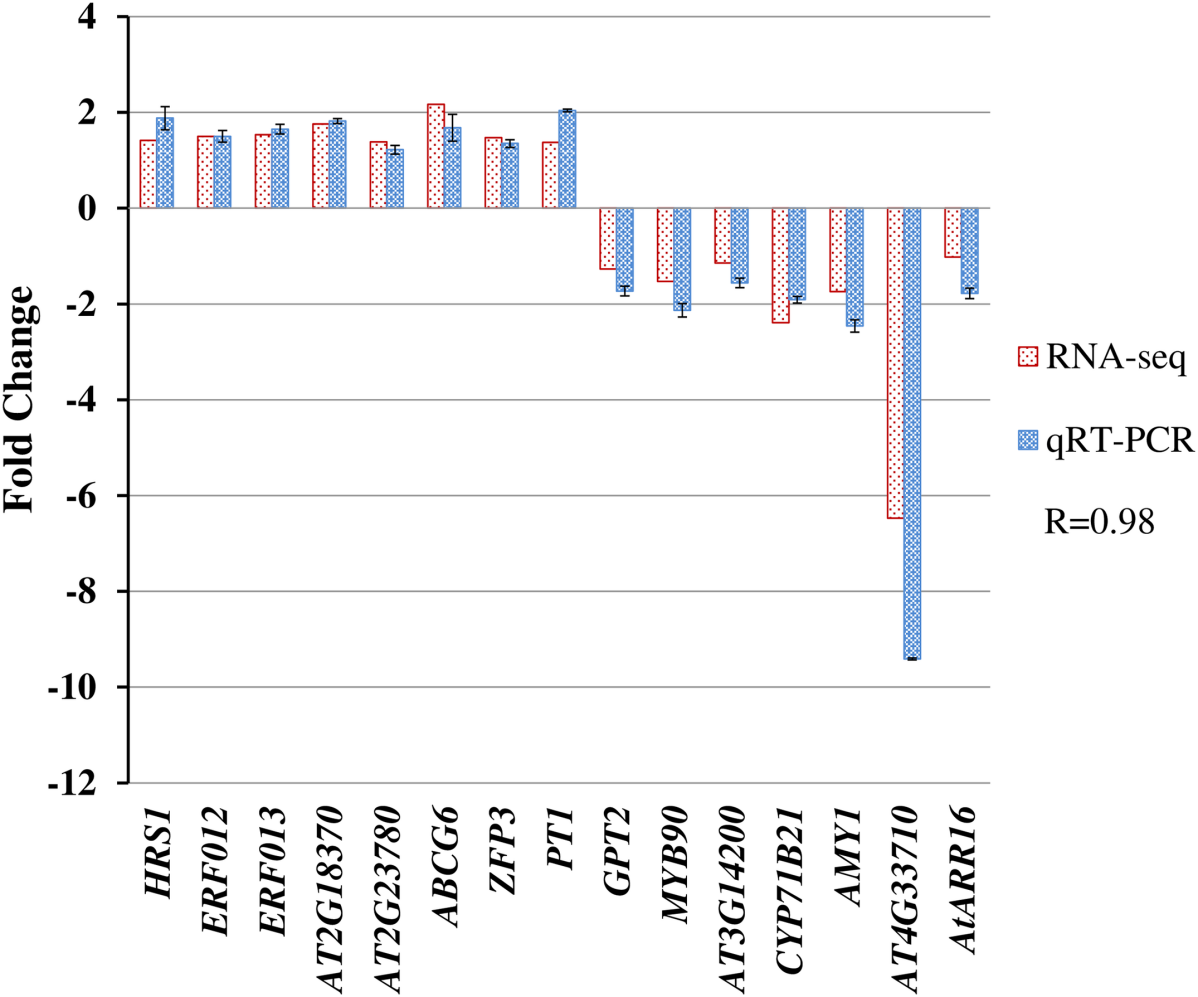
qRT–PCR validation of RNA-Seq results. Fifteen genes were randomly selected from the DEGs (*red columns*) from the RNA-Seq data and were analyzed for differential expression changes (*blue columns*) of the genes. The results were the average of two biological replicate samples in triplicate. *Error bars* indicate the standard error of two biological replicates in qRT–PCR.

To verify the RNA-Seq data, we randomly chose fifteen genes from the DEGs and used Primer Premier software (version 5.0) to design specific primers for the genes (S8 Table). Real time quantitative PCR analyses showed that the relative expression patterns of the genes were consistent with RNA-Seq data, with a correlation coefficient of 0.98 between qRT-PCR and RNA-Seq (Fig 5). The results demonstrated that the RNA-Seq data are reliable.

## Discussion

In the GO classifications of genes, all genes (including the DEGs) were assigned into three main categories that contained 56 annotated functional subcategories (Fig 3A). In this work, we obtained 183 DEGs between the MI(A1) and the MI+ (A2) groups. We confirmed the authenticity of the DEGs data by real time qPCR analyses. The results showed the R value was relatively high (0.98), which proved the RNA-seq data were reliable. GO analyses of up-regulated and down-regulated genes of the DEGs were different, such as the antioxidant activity subcategories (GO:0004601) only presented to MF categories of down-regulated genes. The DEGs are involved in subcategories of biological process (BP) and molecular function (MF) (Fig 3B and 3C). The MF included four different subcategories. They were receptor activity (GO:0004872, GO:0005484), antioxidant activity (GO:0004601), enzyme regulator activity (GO:0046910, GO:0004867) and nutrient reservoir activity (GO:0045735). In the BP group, the number of up-regulated and down-regulated genes was almost the same, but the biological phase subcategory (GO:0048510) was only present in the BP of up-regulated genes. Using KEGG analysis, several DEGs were assigned to KEGG pathways (S4 Table), but most of the DEGs could not be assigned to pathways. Therefore, to better understand the DEGs, we used multiple tools (KEGG, MapMan and DAVID) to analyze the functions and the metabolic pathways of the genes. The results showed that the DEGs were involved in cell wall synthesis, signaling transduction, oxidation-reduction pathways, defense response and secondary metabolisms (S5 and S6 Tables). In Fig 4B, only two genes were assigned to biotic stress pathways. If plant was not attacked by pathogens or pests, these defense genes might not be expressed. Nevertheless, seven DEGs associated with hormones signaling were identified, and the genes were down-regulated in A2. In addition, we also organized the functional classification of DEGs based on previously reported studies (S7 Table). However, multiple DEGs had unknown functions and pathways. For example, PPR family proteins were one of the largest families but also one of the most perplexing in plants. Many PPR proteins were predicted to be targeted to either the mitochondria or the chloroplast [46], and their specific functions were unknown.

The multifunctional MI is emerging as a central feature in plant biochemistry and physiology [4]. MI can be used to generate cell wall polysaccharides [47], which are important precursors of cell wall biosynthesis. MI biosynthesis, the MI oxidation pathway (MIOP) and phytic acid biosynthesis are three major three pathways that are taken into account for MI metabolism analyses [4]. The MIOP is a very important route to balance the concentrations of MI by *myo*-inositol oxygenase (MIOX; E.C. 1.13.99.1), which can maintain normal growth and development in plants [48]. The MIOX is an unique monooxygenase, it catalyzes the conversion of MI into D-glucuronic acid (D-GlcUA), which finally enters the pool of UDP-GlcA and serves as a precursor for plant cell wall polysaccharides. *MIOX2* is a member of the MIOX family and is expressed in almost all tissues of the plant [15]. In our RNA-Seq data, the expression level of the *MIOX*2 gene was up-regulated between A1 and A2, which indicated that additional exogenous MI enhanced the conversion of MI into D-GlcUA in *Arabidopsis*. Another gene of the UDP-GlcA biosynthesis process, UDP-glucose 6-dehydrogenase (AT5G15490, *UGD*3), was also up-regulated in A2. UDP-GlcA biosynthesis includes two pathways, namely the MIOP and SNOP [13]. D-glucose-6-phosphate (D-Glc-6P) is a common precursor in the alternating pathways of MIOP and SNOP. D-Glc-6P is converted, in turn, to phosphoglucomutase by UDP-glucose pyrophosphorylase and UGD (EC 1.1.1.22) and is converted into UDP-GlcA in SNOP. The up-regulated expression levels of *MIOX*2 and *UGD*3 indicated that addition of exogenous MI promotes the oxidation of MI and the biosynthesis of UDP-GlcA. In addition, we observed the expression level of *AtMIOX2* was also upregulated in roots and root calluses of MI+ treatment, respectively (S4A and S4F Fig). We also examined upstream and downstream genes related to MI metabolism, such as *AtMIPS1*, *AtMIPS2*, *AtPIS1*, *AtPLC2*, *AtIPK2β* and *AtIPK1*. These genes were not significantly different in the roots, but *AtMIPS1*and *AtMIPS2* were significantly down-regulated in calluses with MI+ treatment (S4E and S4I Fig). These results demonstrated exogenous MI enhanced the expression of MIOXs, especially in the absence of photosynthetic condition.

In plant cells, the glucose-6-phosphate precursor is mainly a product of starch hydrolysis (e.g., maltose and especially glucose.) [49]. Alpha-amylase (AMYs, EC 3.2.1.1) is essential for starch hydrolysis. We found a gene (AT4G25000, *AMY*1) encoding one of three AMYs in *Arabidopsis*, which was down-regulated in A2. *GPT2* (AT1G61800, Glc-6P/phosphate translocator) was also down-regulated in A2. *GPT*2 acts as a safety valve in situations when carbohydrate metabolism is impaired (i.e., in the absence of transitory starch) or in the presence of increased soluble sugar concentrations [50]. From the KEGG enrichment pathway (S3 Fig; S4 Table) and MapMan pathway (S5 Table) analysis, the plant cell wall formation is closely related to starch and sugar metabolism. Some genes are assigned to both pathways (e.g., *UGD*3). Cell wall biosynthesis is dependent on cell wall polysaccharides. Reversibly glycosylated proteins (RGPs) have been implicated in polysaccharide biosynthesis, and it is speculated that a role in cell wall biosynthesis is likely [51, 52]. Zhao and Liu [51] demonstrated that a cotton RGP gene had a role in noncellulosic polysaccharide biosynthesis of the cell wall. Interestingly, *RGP5* (AT5G16510), a member of the RGP family in *Arabidopsis*, was highly up-regulated in A2. Rautengarten et al. [52] demonstrated ubiquitous expression of *RGP1*, *RGP*2, and *RGP5* throughout plant development, indicative of a general function in plant cell wall metabolism. Additionally, Chen et al. [53] examined a gene related to nuclear export of 60S ribosomal subunits, named *NMD3* (AT2G03820), which affected secondary cell wall thickening and formation of the rough endoplasmic reticulum. We used KEGG and MapMan tools to display a pathway for cell wall metabolism. There were five up-regulated genes related to plant-type cell wall terms in the DEGs (Fig 4; S5 Table). AGP17 (AT2G23130) was a member of Arabinogalactan-proteins, which were localized to plant cell walls or are secreted into the apoplast. We didn’t find *AGP17* gene in the DEGs, but the expression levels of gene were upregulated in roots with MI+ treatment (S4B Fig). AGP17 can impact *Agrobacterium*-mediated transformation efficiency, because it is important both for *Agrobacterium* attachment to roots and for the suppression of host defense responses [54, 55]. These results showed the metabolism of cell wall polysaccharides was affected by exogenous MI. Based on the expression levels of DEGs, we could speculate that MI metabolism is a homeostatic mechanism in plant cells, and cell wall polysaccharide biosynthesis may be one of the methods used to maintain homeostasis. These cell wall compounds might affect *Agrobacterium*-mediated transformation in roots.

As an important molecule, MI participates in numerous life processes. In the past few years, a larger number of studies indicated that MI can impart stress tolerance to plants [10, 20, 22, 23, 56]. Donahue et al. [10] suggested exogenous MI could rescue spontaneous cell death and lesions on leaves in *Arabidopsis atmips1* mutants. In addition, the *atmips1* mutants affected phosphatidylinositol (PtdIns) synthesis and resulted in altered levels of ceramide, a signaling molecule associated with cell death in plant [24, 25]. In our RNA-Seq data, we observed many genes related to immune system processes and cell death (Fig 3). Fig 4A and 4B show the DEGs assigned to the different terms. Fourteen DEGs are associated with stress, including three genes related to biotic stress and eleven genes related to abiotic stress. Interestingly, we found *ESB1* (AT2G28670) and *ABCG6* (AT5G13580) were up-regulated in A2. *ESB1* negatively regulates formation of suberin and plays an essential role in the correct formation of Casparian strips [57]. *ABCG6* is required for synthesis of an effective suberin barrier in roots and seed coats and affects lateral root growth and precocious secondary growth in primary roots. The expression level of *ABCG6* was increased by inducing ABA, IAA, GA_3_, mannitol, and salt stress [58]. These results implied that exogenous MI supplementation may affect the structure of plant cells as well as other biological process (e.g., signaling transduction and hormone metabolism). In terms of abiotic stress, a majority of the DEGs were down-regulated between A1 and A2 (Fig 4B; S5 Table). In addition, MapMan assigned several DEGs into the stress category, and some DEGs are involved in the stress or defense response, such as *SAG13* (AT2G29350), *SCL14* (AT1G07530) and *XERICO* (AT2G04240). *SAG13* was down-regulated in A2. As a senescence-specific marker, *SAG13* expression increases during senescence [59], and *SAG12* and *SAG13* have been widely used to distinguish senescence-related cell death from hypersensitive response-related programmed cell death [60]. The browning/senescing of root calluses (S5A and S5B Fig) indicated that exogenous MI could reduce cell death and maintain normal cell growth and development. *SCL14* is involved in the detoxification of harmful chemicals and encodes a protein that serves as a transcriptional coactivator of TGA transcription [61], and its expression level was up-regulated in A2. Ko et al. [62] demonstrated that the expression of *XERICO* is induced by salt and osmotic stress, and overexpression of this gene is involved in ABA homeostasis. In our work, *XERICO* up-regulation indicated that A2 undergoes more stress than A1. However, we do not know what type of stress was induced and whether it was associated with hormone metabolism. In the experiment, we noticed that supplied with MI in the culture medium resulted in short roots of *Arabidopsis* plants, but without obvious effects on the size of shoots and leaves. Considering the effects of nutrients on root growth, such as phosphate, exogenous supplementary of MI may also affect nutrient absorption.

We also observed several genes that are associated with hormone metabolism, and their expression was down-regulated, except for AT4G23340 (related to the synthesis of gibberellin), between A1 and A2 (Fig 4; S5 Table). The hormones included ethylene, abscisic acid (ABA), salicylic acid (SA) and brassinosteroid (BR) (Fig 4B). ABA and ethylene promote maturation and senescence in plants. These down-regulated genes improve resistance and delay senescence or cell death in plants, and the results are consistent with the *SAG13* results [59, 60]. Using DAVID tools, the vast majority of down-regulated genes were categorized into the oxidation-reduction process (S6 Table). The results indicated that exogenous MI could influence the expression of genes related to hormone metabolism, and may directly or indirectly respond to the various stressors.

Chatterjee and Majumder [29] demonstrated that exogenous *myo*-inositol supplementation prevented salt-induced chromosomal abnormalities and DNA fragmentation, and cell division was restored in the root tip mitotic cells of *Allium cepa*. S6 Table displays three genes associated with chromosomes, termed *SPT16* (AT4G10710), *SUVH1* (AT5G04940) and *SUVH5* (AT2G35160). The SPT16 protein is a subunit of the facilitates chromatin transcription (FACT) complex, and the *SPT16* is expressed in shoots, roots and flowers. FACT assists in transcription elongation of the plant chromatin [63]. In TAIR (http://www.arabidopsis.org) annotations, *SPT16* is also involved in DNA repair and regulation of transcription. *SUVH1* and *SUVH5* are two of ten Su(var)3-9 homologues in *Arabidopsis*. They are associated with heterochromatin, possess histone methyltransferase activity [64], and participate in the organization of transcriptionally repressive chromatin [65]. *SUVH5* contributes to the maintenance of H3 mK9 and CMT3-mediated non-CG methylation *in vivo*, and it can also methylate *Arabidopsis* histone H2A variants *in vitro* [66]. H2A variants are involved in telomeric silencing [67], protecting euchromatin from heterochromatin spreading [68], and the nonhomologous end-joining DNA repair pathway [69]. We tested the expression levels of *AtSUVH1* and *AtSUVH5* in roots and root calluses. The results indicated exogenous MI can promote the expression of the two genes, and the expression of *AtSUVH1* in root increased more than that in root calluses (S4C, S4D, S4G and S4H Fig). The results suggested that exogenous MI might affect modifying factors of chromatin. Additionally, a DNA repair gene, AT3G50880, was up-regulated in A2 (Fig 4; S5 Table). Mysore et al. [70] showed that histone H2A played a role in *Agrobacterium* transformation. Moreover, some genes related to chromosome modification and DNA repair were important factors for T-DNA integration into chromatin [71, 72]. We performed *Agrobacterium*-mediated *Arabidopsis* root transformation, and the results showed that the transformation efficiency was significantly different between the MI- and MI+ treatments (S1C and S1D Fig). The results indicated that exogenous MI might enhance T-DNA integration into chromatin [30]. Naumann et al. [64] suggested that subtle changes in chromatin structure might be required for fine-tuning of gene expression, and for this reason, multi-gene families for DNA and histone modification systems were found in plants. Our data showed that exogenous MI added to culture medium might impact regulation of transcription for chromosome structure.

As a small molecule, MI’s role in the plant cell osmotic regulation remains unclear. Nevertheless, we observed several genes related to substance transport by MapMan analysis. For instance, *NRT2*.*1* (AT1G08090), *PT1* (AT5G43350), *GPT2*, *EMB1513* (AT2G37920), *ABCG6*, proton-dependent oligopeptide transport family protein (AT1G72140), and carbohydrate transmembrane transporter (AT2G18590) (S5 Table) encode proteins involved in intracellular and intercellular transport. These genes were associated with the absorption of nitrate [73] and phosphorus [74, 75], sugar signaling [76] and ion transmembrane transport [77]. The phosphorus is one of key elements in the growth and development of plant. *PT1* influenced phosphorus uptake [74]. When grown under limited phosphate (P) conditions, *A*. *thaliana* plants show dramatic changes in root architecture, including a reduction in primary root length, increased formation of lateral roots and greater formation of root hairs [78]. In our study, similar phenomena were observed in roots of MI+ treatment (S2 Fig). We speculated that the increased formation of lateral roots might be a response strategy that plants used to extend the nutrition uptake area in the presence of exogenous MI, and primary root growth might be affected by exogenous MI. The higher *Agrobacterium*-mediated transformation efficiency in MI+ treatment might be related to the more lateral roots and root hairs. They might increase the number of *Agrobacterium* cells attaching to the roots due to the increased root surface. In addition, *GPT2* can maintain the balance between the production of carbohydrates [78], and EMB1513 is involved in copper ion transmembrane transport [77]. The results showed that exogenous MI might act as a regulator to participate in the transportation of carbohydrates and other substances, and the altered transcription levels were a response whereby plant cells adapted to environmental conditions to maintain normal growth and development.

In conclusion, this work was the first report about transcriptional analysis of *Arabidopsis* with exogenous MI treatments by RNA-Seq method. We found that exogenous MI affected the growth and development of *Arabidopsis*. In the DEGs, we observed many important genes related to the synthesis of cell wall, stress response, chromosome modification, substance transport, oxidation-reduction reaction and hormone regulation were differently expressed. These results suggested exogenous MI might act as a signal molecule affecting the process of cell metabolism in plants and might also influence the metabolism of nucleic acids and chromosome modification and even cause the morphological changes in the process of growth and development. These changes may cause differences in *Agrobacterium*-mediated transformation efficiency between MI- and MI+ treatments. Thus, exogenous MI as an exogenous regulator could be applied in genetic engineering of plant.

## Supporting Information

S1 Fig Growth and transformation efficiency of roots in *Arabidopsis thaliana* ecotype Col-0 by exogenous *myo*-inositol (MI) treatments were affected. The phenotype of roots growth (**A**) and the primary roots length (**C**) at 27 days; the tumorigenesis phenotype (**B**) and transformation efficiency (**D**) of 4 weeks old *A*. *thaliana* Col-0 roots affer transformed 4 weeks. MI+0 represent without exogenous MI treatment. MI+50, MI+100, MI+500 and MI+1000 represent adding 50, 100, 500 and 1000 mg l^-1^ exogenous MI treatments in medium, respectively. The plant growth conditions and *Agrobacterium*-mediated roots transformation protocols as Gelvin (2006). The root length and Transformation efficiency of root segments statistic used *ANOVA* method. The lowcase letters indicate significant differences among treatments (*P*<0.05, the number of roots = 30; the numner of root segments >360, mean ± SE). Root length was average value that 30 primary roots per treatment were measured. Transformation efficiency = tumorigenesis root segments /total root segments ×100%. We used low concentration (OD_600_ = 0.080 Abs, resuspended *Agrobacterium* strains in 0.9% NaCl at 5×10^7^ cfu ml^-1^) of *A*. *tumefaciens* A208 (for tumorigenesis assays) to transformed root segments. The bacteria infected the root segments for 5 min. The bacteria and the root segments were cocultured for 48 h under dark in a growth chamber at 22°C. After cocultivation, the root segenents was rinsed with Timetin solution (100 mg l^-1^) to kill the bacteria. The infected root segments were separated into individual root segments, which were cultured for 4 weeks under 16 h light and 8 h dark in a growth chamber at 25°C. The statistical analysis of transformation were performed at 4 weeks.
(TIF)

S2 Fig The number of lateral roots and root hairs phenotype with and without exogenous MI treatments. MI+0 (MI-) represent no adding exogenous MI treatment; MI+100 (MI+) represent adding 100 mg l^-1^ exogenous MI treatment. **A** and **C** indicated the middle of taproot. **B** and **D** indicated the root tip. **E** indicated the number of lateral roots per primary root in with (MI+100) or without (MI+0) exogenous MI treatments (Significant difference at *P*<0.05, compared with without MI treatment (MI-) by Student’s *t* test. n = 30 primary roots, mean ± SE). Stereoscopic microscope was used to observe the roots and root hairs under same magnification.
(TIF)

S3 Fig KEGG pathways of the DEGs. The 32 genes of all 183 DEGs are assigned to 32 KEGG pathways. The ordinates represent the KEGG pathways (*left*) and function classifications of KEGG enrichment (*right*). The abscissa represents the percentage that the number of genes in each pathway divided by the total assigned genes of KEGG.
(TIF)

S4 Fig Analysis of the expression levels of *MIOX2*, *SUVH1*, *SUVH5*, *AGP17*, *MIPS*, *PIS1*, *PLC2*, *IPK2β* and *IPK1* genes in roots and root calluses. **A, B, C, D** and **E** were the relative expression levels of genes in root by qRT-PCR (n = 3, means ± SDS). **F, G, H** and **I** were the relative expression levels of genes in one-month callus by qRT-PCR. *, significant difference at *P*<0.05, compared with without MI treatment (MI-) by Student’s *t* test. (n = 3; means ± SDS). Total RNA was extracted using the TRIzol^®^ reagent (Invitrogen) according to the manufacturer’s protocol. The internal reference gene were *AtElf4a*. The primers were in S9 Table.
(TIF)

S5 Fig Calluses derived from root of *Arabidopsis thaliana* ecotype Col-0 wild type. Root-calluses of *Arabidopsis thaliana* ecotype Col-0 wild type were cultured on B5 callus inducing medium with and without *myo*-inositol supplementation when root segments were growth for 28 days. **A**: B5 media without *myo*-inositol supplementation; **B**: B5 media with100 mg l^-1^ *myo*-inositol supplementation. B5 callus inducing medium containing 3.1 g l^-1^ B5 minimal salts minimal salts (Gibco), 0.5 g l^-1^ MES, 1 ml l^-1^ vitamin stock solution (1000X, 0.5 mg ml^-1^ nicotinic acid, 0.5 mg ml^-1^ pyridoxine, 0.5 mg ml^-1^ thiamine-HCl.), 20 g l^-1^ glucose, 1 ml l^-1^ indole-3-acetic acid (IAA) stock solution(1000X, 5 mg ml^-1^ in H_2_O.), 0.5 ml l^-1^ 2,4-dichlorophenoxy acetic acid (2,4-D) stock solution (2000X, 1 mg ml^-1^ in H_2_O), 0.5 ml l^-1^ Kinetin stock solution (2000X, 0.6 mg ml^-1^ in H_2_O). Adjust PH to 5.7 with 1 N KOH; add 7.5 g l^-1^ Bacto agar. Autoclave for no more than 20 min. The roots were cutted about 0.5 cm segments and put a single root on the B5 callus inducing medium, each Petri dish placed about 20 roots. Seal the Petri dishes with parafilm, and culture roots for 28 days in a growth chamber at 25°C and in dark. When 28 days, the color and size of calluses were significant differences (The calluses were browning and small in without *myo*-inositol treatment, but the calluses were wihte and big in with *myo*-inositol treatment).
(TIF)

S1 Table A total of 21868 genes derived from two cDNA libraries. (XLS)

S2 Table List of the 184 new genes detected from the two cDNA libraries. (XLS)

S3 Table List of the 183 differentially expressed genes (DEGs) between the two treatments with *myo*-inositol in *Arabidopsis thaliana*. (XLS)

S4 Table Overview of all 32 KEGG pathways. (XLS)

S5 Table Details of all 52 branch pathways in the 21 MapMan major pathways. (XLS)

S6 Table The list of functional annotation clustering by DAVID analysis. (XLSX)

S7 Table The functional groups of some major genes reported in plants. (XLSX)

S8 Table Primers used in the real-time qRT-PCR analysis for validation of RNA-Seq results. (XLS)

S9 Table Primers used in the real-time qRT-PCR analysis for *MIOX2*, *SUVH1*, *SUVH5*, *AGP17*, *MIPS*, *PIS1*, *PLC2*, *IPK2β* and *IPK1* genes in roots and root calluses. (XLS)
