# Supplementary figures and images for: Transcriptomic Profiling Analysis of Arabidopsis thaliana Treated with Exogenous Myo-Inositol
Source: PLoS One. 2016 Sep 7;11(9):e0161949. doi: 10.1371/journal.pone.0161949 (PMC5014391; doi:10.1371/journal.pone.0161949)

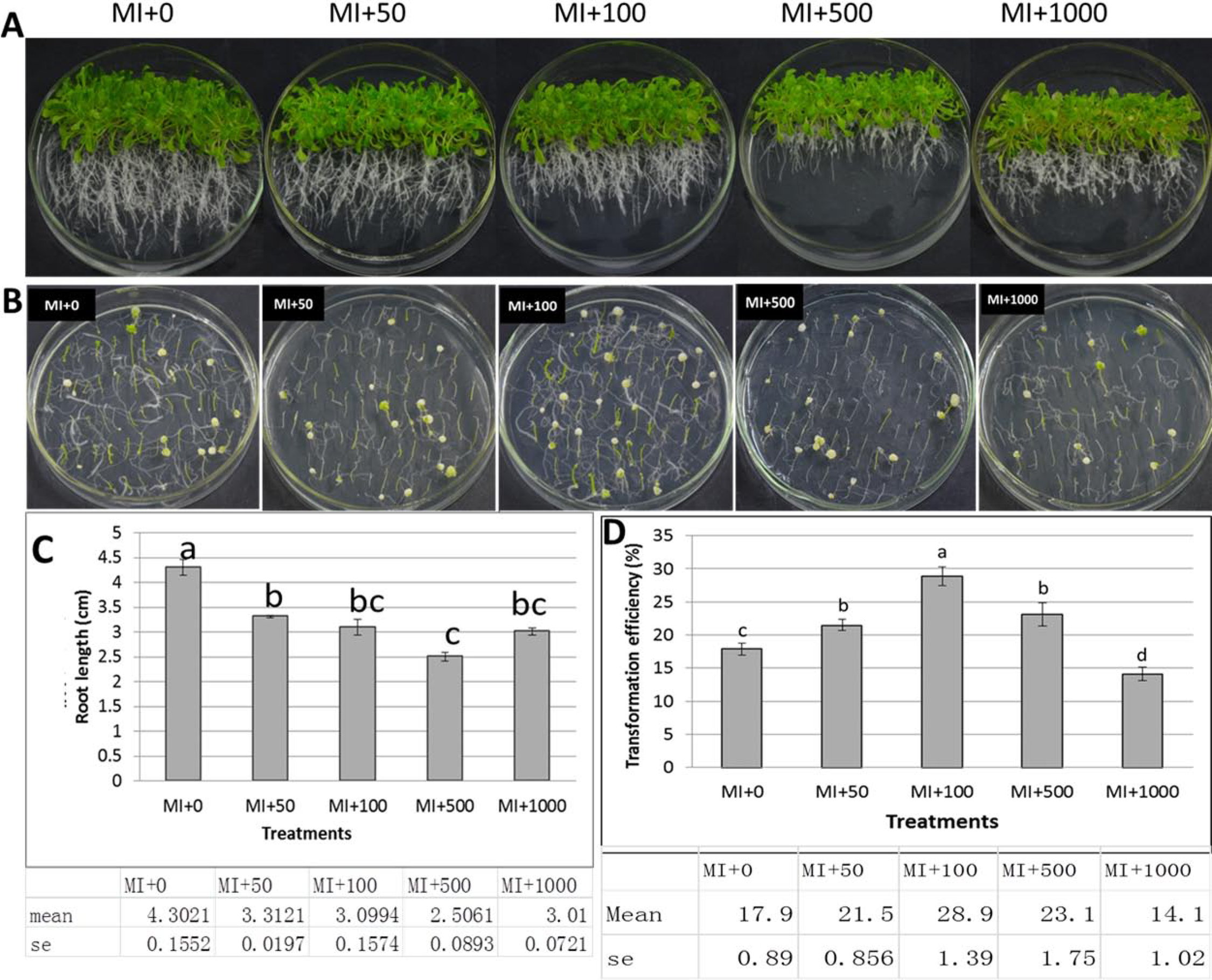

Supplement: S1 Fig — The phenotype of roots growth (A) and the primary roots length (C) at 27 days; the tumorigenesis phenotype (B) and transformation efficiency (D) of 4 weeks old A. thaliana Col-0 roots affer transformed 4 weeks. MI+0 represent without exogenous MI treatment. MI+50, MI+100, MI+500 and MI+1000 represent adding 50, 100, 500 and 1000 mg l-1 exogenous MI treatments in medium, respectively. The plant growth conditions and Agrobacterium-mediated roots transformation protocols as Gelvin (2006). The root length and Transformation efficiency of root segments statistic used ANOVA method. The lowcase letters indicate significant differences among treatments (P<0.05, the number of roots = 30; the numner of root segments >360, mean ± SE). Root length was average value that 30 primary roots per treatment were measured. Transformation efficiency = tumorigenesis root segments /total root segments ×100%. We used low concentration (OD600 = 0.080 Abs, resuspended Agrobacterium strains in 0.9% NaCl at 5×10^7^ cfu ml-1) of A. tumefaciens A208 (for tumorigenesis assays) to transformed root segments. The bacteria infected the root segments for 5 min. The bacteria and the root segments were cocultured for 48 h under dark in a growth chamber at 22°C. After cocultivation, the root segenents was rinsed with Timetin solution (100 mg l-1) to kill the bacteria. The infected root segments were separated into individual root segments, which were cultured for 4 weeks under 16 h light and 8 h dark in a growth chamber at 25°C. The statistical analysis of transformation were performed at 4 weeks. (TIF) [file pone.0161949.s001.tif]

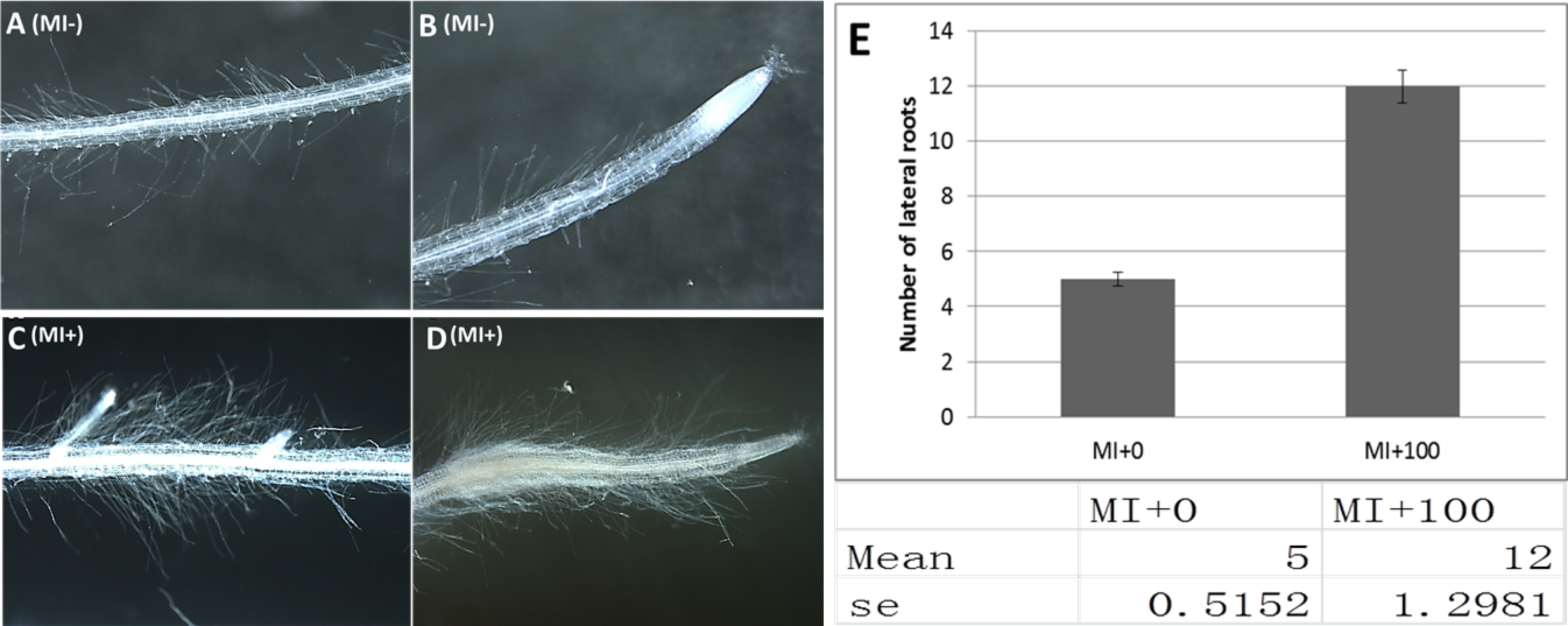

Supplement: S2 Fig — MI+0 (MI-) represent no adding exogenous MI treatment; MI+100 (MI+) represent adding 100 mg l-1 exogenous MI treatment. A and C indicated the middle of taproot. B and D indicated the root tip. E indicated the number of lateral roots per primary root in with (MI+100) or without (MI+0) exogenous MI treatments (Significant difference at P<0.05, compared with without MI treatment (MI-) by Student’s t test. n = 30 primary roots, mean ± SE). Stereoscopic microscope was used to observe the roots and root hairs under same magnification. (TIF) [file pone.0161949.s002.tif]

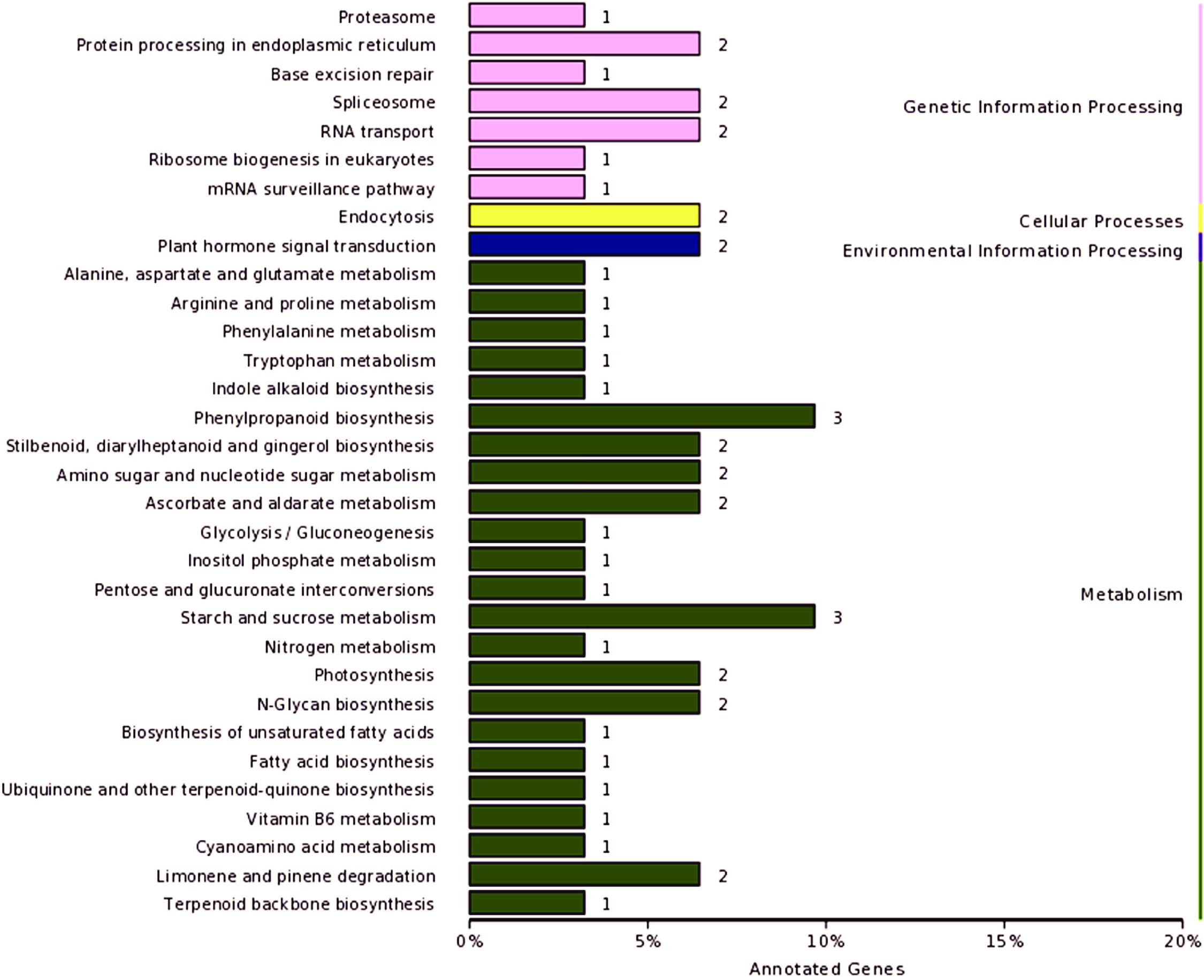

Supplement: S3 Fig — The 32 genes of all 183 DEGs are assigned to 32 KEGG pathways. The ordinates represent the KEGG pathways (left) and function classifications of KEGG enrichment (right). The abscissa represents the percentage that the number of genes in each pathway divided by the total assigned genes of KEGG. (TIF) [file pone.0161949.s003.tif]

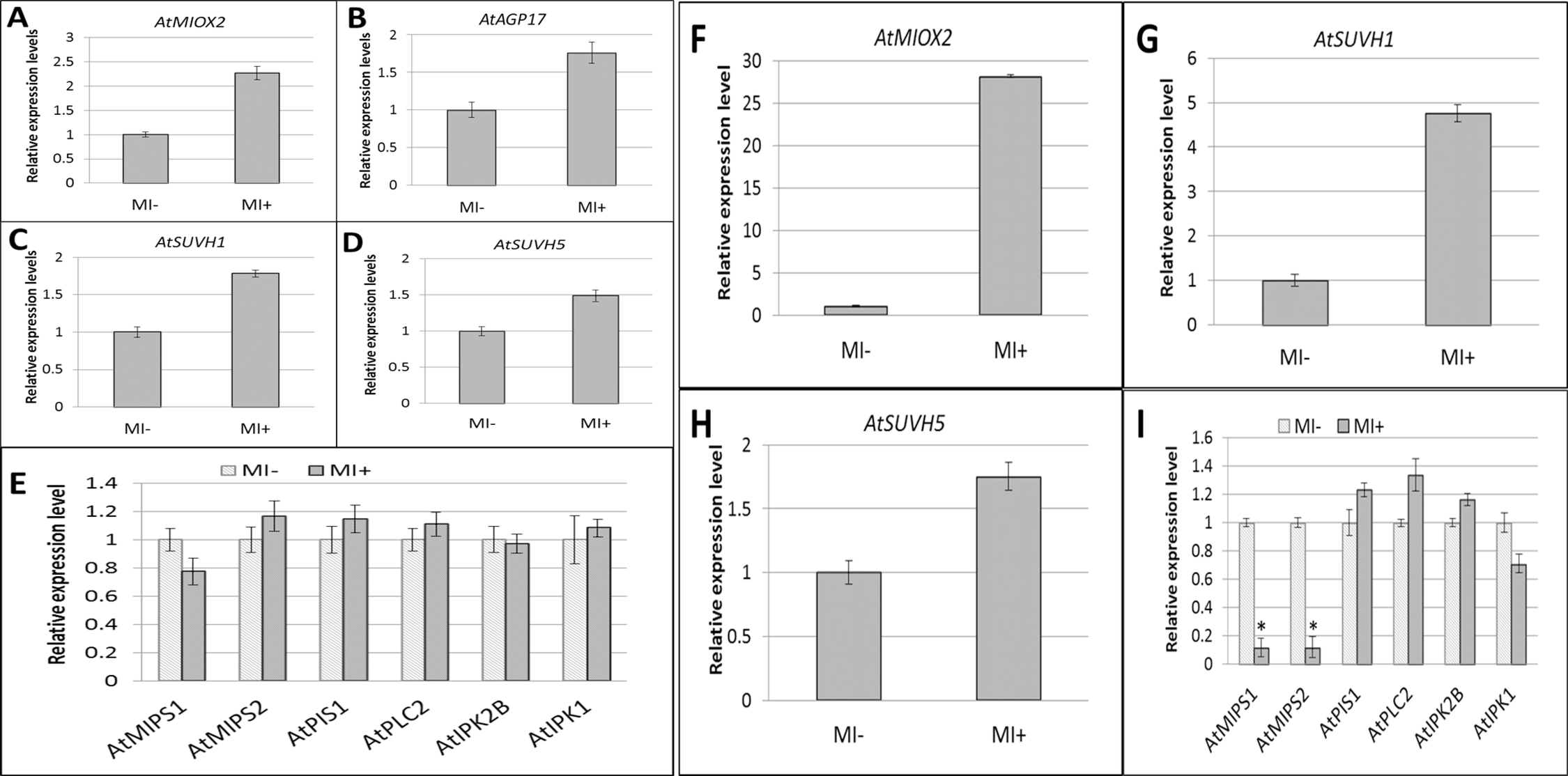

Supplement: S4 Fig — A, B, C, D and E were the relative expression levels of genes in root by qRT-PCR (n = 3, means ± SDS). F, G, H and I were the relative expression levels of genes in one-month callus by qRT-PCR. *, significant difference at P<0.05, compared with without MI treatment (MI-) by Student’s t test. (n = 3; means ± SDS). Total RNA was extracted using the TRIzol® reagent (Invitrogen) according to the manufacturer’s protocol. The internal reference gene were AtElf4a. The primers were in S9 Table. (TIF) [file pone.0161949.s004.tif]

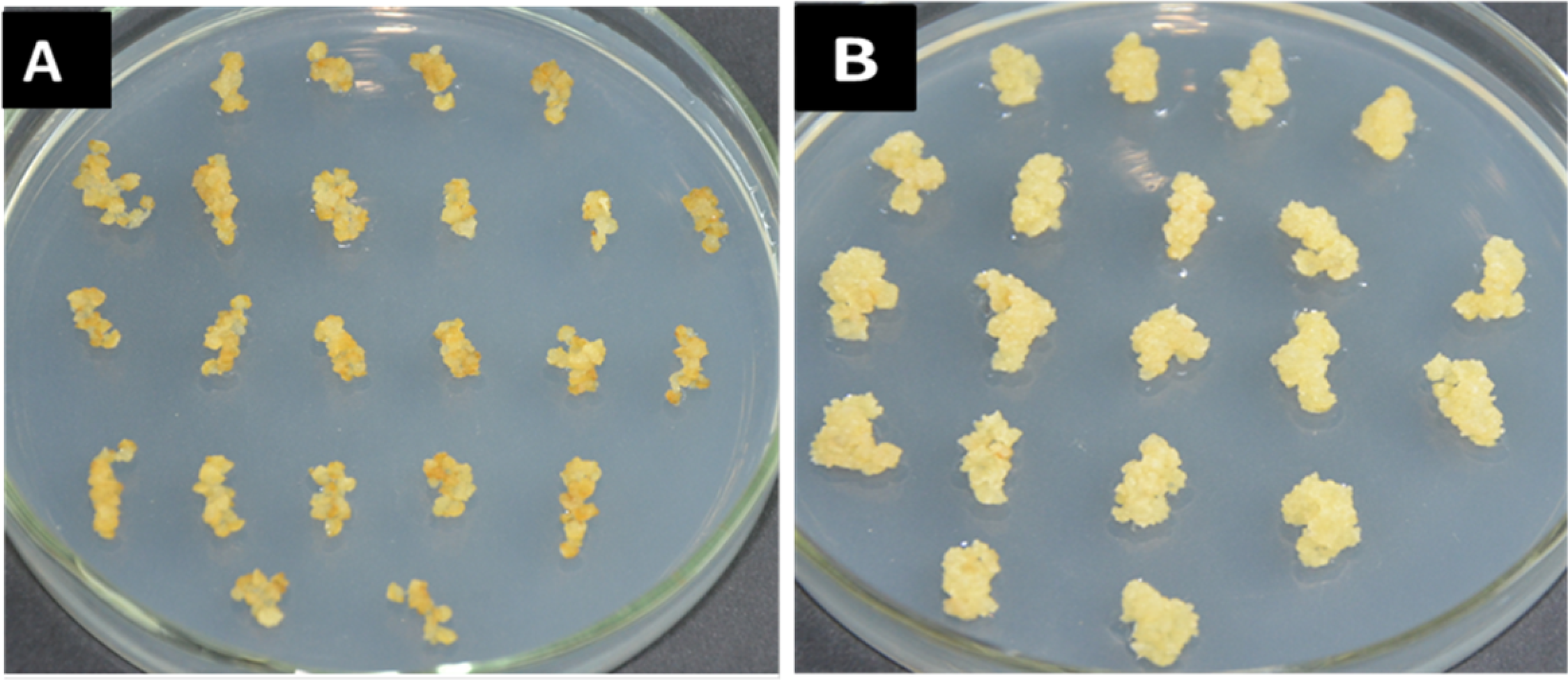

Supplement: S5 Fig — Root-calluses of Arabidopsis thaliana ecotype Col-0 wild type were cultured on B5 callus inducing medium with and without myo-inositol supplementation when root segments were growth for 28 days. A: B5 media without myo-inositol supplementation; B: B5 media with100 mg l-1 myo-inositol supplementation. B5 callus inducing medium containing 3.1 g l-1 B5 minimal salts minimal salts (Gibco), 0.5 g l-1 MES, 1 ml l-1 vitamin stock solution (1000X, 0.5 mg ml-1 nicotinic acid, 0.5 mg ml-1 pyridoxine, 0.5 mg ml-1 thiamine-HCl.), 20 g l-1 glucose, 1 ml l-1 indole-3-acetic acid (IAA) stock solution(1000X, 5 mg ml-1 in H2O.), 0.5 ml l-1 2,4-dichlorophenoxy acetic acid (2,4-D) stock solution (2000X, 1 mg ml-1 in H2O), 0.5 ml l-1 Kinetin stock solution (2000X, 0.6 mg ml-1 in H2O). Adjust PH to 5.7 with 1 N KOH; add 7.5 g l-1 Bacto agar. Autoclave for no more than 20 min. The roots were cutted about 0.5 cm segments and put a single root on the B5 callus inducing medium, each Petri dish placed about 20 roots. Seal the Petri dishes with parafilm, and culture roots for 28 days in a growth chamber at 25°C and in dark. When 28 days, the color and size of calluses were significant differences (The calluses were browning and small in without myo-inositol treatment, but the calluses were wihte and big in with myo-inositol treatment). (TIF) [file pone.0161949.s005.tif]
